# Medical Corps of the Confederate Army and Navy, 1861-1865

**Published:** 1890-08

**Authors:** Joseph Jones

**Affiliations:** New Orleans, Louisiana, Surgeon-General United Confederate Veterans


### MEDICAL CORPS OF THE CONFEDERATE ARMY AND NAVY, 1861–1865.

---

### OFFICIAL REPORT OF JOSEPH JONES, M. D., OF NEW ORLEANS, LOUISIANA, SURGEON-GENERAL UNITED CONFEDERATE VETERANS.

---

156 WASHINGTON AVENUE,
NEW ORLEANS, LA., June 30th, 1890.

*His Excellency John B. Gordon, General Commanding United Confederate Veterans, Atlanta, Georgia:*

GENERAL—I have the honor to submit the following:

The Medical Department of the Confederate States was a branch of the War Department, and was under the immediate supervision of the Secretary of War.

The Surgeon-General of the Confederate States was charged with the administrative details of the Medical Department, the government of hospitals, the regulation of the duties of surgeons and assistant-surgeons, and the appointment of acting medical officers where needed, for local or detached service. He issued orders and instructions relating to professional duties of medical officers, and all communications from them which required his action were made directly to him.

The great struggle for the independence of the Confederate States ended twenty-five years ago, and all soldiers in the Confederate army, from the commanding general to the private in the ranks, were, by the power of the conquering sword, reduced to one common level, that of the parolled prisoner of war. The objects of the association of the United Confederate Veterans of 1890 are chiefly historical and benevolent. We conceive, therefore, that the labors of the Surgeon-General relate to two important objects:

FIRST. *The collection and preservation of the records of the medical corps of the Confederate army and navy.*

SECOND. *The determination, by actual investigation and inquiry, the numbers and condition of the surviving Confederate soldiers who have been disabled by wounds and disease, received in their heroic defense of the rights and liberties of the Southern States.*

To accomplish the first object the following Circular No. 1 has been issued:

I. *The collection and preservation of the records of the medical officers of the Confederate army and navy.*

(CIRCULAR NO. 1.)

OFFICE SURGEON-GENERAL U. C. V.,
156 WASHINGTON AVENUE,
NEW ORLEANS, LA., April 9th, 1890.

*To the Survivors of the Medical Corps of the Confederate States Army and Navy.*

COMRADES—The surrender of the Army of Northern Virginia on this day twenty-five years ago, practically ended the struggle for the independence of the Southern States; and during this quarter of a century death has thinned our ranks and our corps can now oppose but a broken line in the great struggle against human suffering, disease and death. S. P. Moore, Surgeon-General of the Confederate army, is dead; Chas. Bell Gibson, Surgeon-General of Virginia, Surgeons L. Guild, A. J. Ford, J. H. Berrien, J. T. Darby, W. A. Carrington, S. A. Ramsey, Samuel Choppin, Robert J. Breckenridge, E. N. Covey, E. S. Gaillard, E. A. Flewellen, Paul F. Eve, O. F. Manson, Louis O. Foard, S. E. Habersham, James Bolton, Robert Gibbes, and a host of other medical officers of the Confederate States army are dead.

The Association of the United Confederate Veterans was formed in New Orleans in 1889, the objects of which are historical, social and benevolent. Our illustrious commanding General, John B. Gordon, of Georgia, has ordered the United Confederate Veterans to assemble in Chattanooga, Tennessee, on July 3d, 1890. It is earnestly hoped that every surviving member of the medical corps of the Confederate army will meet upon this important occasion, and promote by his presence and his counsels the sound interests of the United Confederate Veterans.

It is of the greatest importance to the future historian, and also to the honor and welfare of the medical profession of the South, that careful records should be furnished to the Surgeon-General of the United Confederate Veterans, embracing the following data:

I. Name, age, nativity, date of commission in the Confederate States army, nature and length of service of each and every member of the medical corps of the Confederate States army.

II. Obituary notices and records of all deceased members of the medical corps of the Confederate army.

III. The titles and copies of all field and hospital reports of the medical corps of the Confederate army.

IV. Titles and copies of all published and unpublished reports relating to military surgery and to diseases of armies, camps, hospitals and prisons.

The object proposed to be accomplished by the Surgeon-General of the United Confederate Veterans is the collection, classification, preservation and final publication, of all the documents and facts relating to the history and labors of the medical corps of the Confederate States army during the civil war, 1861-1865. Everything which relates to this critical period of our national history which shall illustrate the patriotic, self-sacrificing and scientific labors of the medical corps of the Confederate States army, and which shall vindicate the truth of history, should be industriously collected, filed and finally published. It is believed that invaluable documents are scattered over the whole land, in the hands of the survivors of the civil war of 1861-1865, which will form material for the correct delineation of the medical history of the corps which played so important a part in the great historic drama.

Death is daily thinning our ranks, whilst time is laying its heavy hands upon the heads of those whose hair is already whitening with the advance of years and the burden of care. No delay, fellow-comrades, should be suffered in the collection and preservation of these precious documents.

To this task of collecting all documents, cases, statistics and facts relating to the medical history of the Confederate army, the Surgeon-General of the United Confederate Veterans invites the immediate attention and co-operation of his comrades and compatriots throughout the South.

Respectfully, your obedient servant,

JOSEPH JONES, M. D.,

*Surgeon-General United Confederate Veterans.*

### FORMATION OF THE MEDICAL CORPS OF THE CONFEDERATE ARMY AND NAVY.

The entire army of the Confederate States was made up of volunteers from every walk in life, and the surgical staff of the army was composed of general practitioners from all parts of the Southern country, whose previous professional life, during the period of unbroken peace which preceded the civil war of 1861-

1865, gave them but little surgery, and very seldom presented a gun-shot wound.

The story of the hygiene of the vast armies, hastily collected to repel invasion, poorly equipped and scantily fed, as well as the frightful experience of the wounded upon the battle-field, and the horrible sufferings of the sick and wounded in the hospitals, unfolded a vast field for the exercise of the highest skill and the loftiest patriotism of the medical men of the South.

This body of men, devoted solely to the preservation of the health of the troops in the field, to the succor of the wounded on the battle-field, and the preservation of their precious lives, and the surgical care of their mangled bodies and limbs, and the treatment of their diseases in field and general hospital, responded to every call of their bleeding country, and formed upon land and upon sea one indivisible corps, which penetrated all arms of the service, and labored for every soldier, however exalted or however low his rank.

When the storm of war suddenly broke upon the Confederacy, and the thunders of cannon were heard around her borders, and her soil trembled with the march of armed battalions; when her ports were blockaded, and medicines and surgical instruments and works were excluded as "contraband of war," the medical practitioners of the South gave their lives and fortunes to their country, and without any prospect of military or political fame or preferment. They searched the fields and forests for remedies; they improvised their surgical instruments from the common implements of every-day life; they marched with the armies, and watching by day and night in the trenches, the Southern surgeons rescued the wounded on the battle-field, binding up the wounds and preserving the shattered limbs of their countrymen. The southern surgeons, through four long years, opposed their skill and tireless energies to the ravages of war and pestilence. At all times, and under all circumstances, in rain or in sunshine, in the cold of winter and in the burning heat of summer, amid the roar of battle and the hissing of bullets, the shriek and crash of shells, the brave hearts, cool heads and strong arms of the Southern surgeons were employed but for one purpose—the preservation of the health, the lives and the limbs of their countrymen. The Southern surgeons were the first to succor the wounded and the sick, and their ears recorded the last messages of love and affection for country and kindred, and their hands closed the eyes of the dying Confederate soldiers.

When the sword decided the cause against the South and the men who had for four years borne the Confederacy upon their bayonets, surrendered prisoners of war, the members of the medical corps of the Confederate army and navy returned to their desloated homes, and resumed the practice of their profession, spoke words of cheer to their distressed countrymen, administered to the sick and wounded Confederate soldiers, and extended their noble and disinterested charities to the widows and orphans of the bereaved and distressed country.

Whilst the political soldiers rose to wealth and power upon the shoulders of the sick and disabled Confederate army, by sounding upon all occasions "HIS WAR RECORD," the modest veterans of the medical corps of the Confederate army and navy were content to serve their sick and wounded and distressed comrades, asking and receiving no other reward than that "peace which passeth all understanding," which flows from the love of humanity, springing from a generous and undefiled heart. It is but just and right that a Roll of Honor should be formed of the band of medical heroes and veterans.

MAGNITUDE OF THE LABORS OF THE MEDICAL CORPS OF THE CONFEDERATE ARMY AND NAVY.

Some conception of the magnitude of the labors performed in field and hospital service by the officers of the medical corps of the Confederate army may be formed by the consideration of the following general results:

KILLED, WOUNDED AND PRISONERS OF THE CONFEDERATE ARMY, 1861-1865.

| Year. | Killed. | Wounded. | Prisoners. |
|---|---|---|---|
| 1861 | 1,315 | 4,054 | 2 772 |
| 1862 | 18,582 | 68,659 | 48,300 |
| 1863 | 11,876 | 51,313 | 71,211 |
| {1664 / 1865} | 22,000 | 70,000 | 80,000 |
| Total.................. | 53,773 | 194,226 | 202,283 |

During a period of nineteen months, January, 1862,—July, 1863, inclusive, over one million cases of wounds and disease were entered upon the Confederate field reports, and over four hundred thousand cases of wounds and disease upon the hospital reports. The number of cases of wounds and disease treated in the Confederate field and general hospitals was even greater during the following twenty-two months, ending April, 1865. It is safe to affirm therefore, that more than three million cases of wounds and disease were cared for by the officers of the medical corps of the Confederate army during the civil war of 1861-65.

The figures of course do not indicate that the Confederacy had in the field an army approaching three million and a half. On the contrary, the Confederate forces actively engaged during the war, 1861-65, did not exceed 600,000. Each Confederate soldier was on an average disabled for a greater or lesser period, by wounds and sickness, about six times during the war.

LOSSES OF THE CONFEDERATE ARMY, 1861-65.

Confederate forces actively engaged during the war 1861-65, 600,000. Grand total of deaths from battle, wounds and disease, 200,000. Loss of the Confederate army in prisoners during the war on account of the policy of non-exchange adopted and enforced by the United States, 200,000. Losses of the Confederate army from discharge for disability from wounds and disease, and from desertion during the war 1861-65, 100,000.

If this calculation be correct one-third of all the men actively engaged on the Confederate side was either killed outright upon the field, or died of disease or wounds. Another third of the entire number was captured and held for indefinite periods, prisoners of war ; and of the remaining two hundred thousand, at least one-half was lost to the service by discharges and desertion. At the close of the war, then, the available armed force in the field and fit for duty numbered scarcely one hundred thousand men. The great army of Northern Virginia, surrendered by General Robert E. Lee, on the 9th of April, 1865, could not muster ten thousand men of all arms fit for active warfare.

Of this body of 600,000 men, 53,773 were killed outright, and

194,026 wounded on the battle-field. One-third of the entire Confederate army was confided to the Confederate surgeons for the treatment of battle wounds, and in addition to such gigantic services, the greater portion, if not the entire body, of the 600,-000 men were under the care of the medical department for the treatment of disease. Well may it be said that to the surgeons of the medical corps is due the credit of maintaining this host of troops in the field.

Such records demonstrate beyond dispute the grand triumphs and glory of medicine, proving that if the physician be the preserver of nations in time of peace, he is no less the preserver and defender of armies during war. These records show that the medical profession, however indispensable in the economy of government during peace, becomes the basis of such economy during war. These statistics prove the importance of medicine and its glorious triumphs, and elevate it logically to its true position in the estimation of not only the physicians, but in that also of the warrior and statesman. The energy and patriotic bravery of the Confederate soldier are placed in a clear light when we regard the vast armies of the Federals to which they were opposed. The whole number of troops mustered into the service of the Northern army during the war of 1861–65 was 2,789,893, or about three times as large as the entire fighting population of the Southern Confederacy.

At the time of the surrender of the Confederate armies, and the close of active hostilities, the Federal force numbered 1,000,-516 of all arms, officers and men, and equalled in numbers the entire fighting population of the Southern Confederacy. Opposed to this immense army of one million of men, supplied with the best equipments and arms, and wtih the most abundant rations of food, the Confederate government could oppose less than one hundred thousand war-worn and battle-scarred veterans, almost all of whom had at some time been wounded, and who had followed the desperate fortunes of the Confederacy for four years, with scanty supplies of clothing, with coarse and scant rations, and almost without pay. Yet the spirit of the Confederate soldier remained proud and unbroken to the last charge, as was conclusively shown by the battles of Franklin and Nashville, Tenn.; the operations around Richmond and Petersburg; the last charge of the army of Northern Virginia, the defense of Fort McAllister, on the Ogeechee river in Georgia, where 200 Confederate soldiers, in an open earthwork, resisted the assault of more than 5,000 Federal troops, and never surrendered, but were cut down at their guns; at West Point, Ga., where there was a similar disparity between the garrison and the assaulting corps, where the first and second in command were killed and the Confederates cut down within the fort; the defense of Mobile in Alabama, and the battle of Bentonville in North Carolina.

### NUMBER OF OFFICERS AND ROSTER OF THE MEDICAL CORPS OF THE CONFEDERATE ARMY AND NAVY.

The destruction by fire of the medical and surgical records of the Confederate States deposited in the Surgeon-General's office in Richmond, Va., in April 1865, has rendered this, the preparation of a complete roster of the medical corps, very difficult, if not impossible.

A general estimate of the aggregate number of medical officers employed in the Medical Department of the Southern Confederacy may be determined by the number of commissioned officers in the Confederate army down to the rank of lieutenant-colonel. Each regiment in the Confederate army was entitled to one colonel, one surgeon and one or two assistant surgeons. A medical officer was generally attached to each battalion of infantry, cavalry or artillery. General, major, lieutenant-general, major-general and brigadier-generals frequently, if not always, had attached to their staff medical doctors, inspectors, and surgeons of corps, divisions and brigades.

We gather the following figures from the elaborate and invaluable "roster of general officers, etc., in the Confederate service" by Chas. C. Jones, Jr., Augusta, Ga., prepared from official sources.

| | |
|---|---|
| Confederate States Army— | |
| Generals | 6 |
| Provisional Army— | |
| Generals | 2 |
| Confederate States Army, Regular and Provisional— | |
| Lieutenant-Generals | 21 |
| Major-Generals | 99 |
| Brigadier-Generals | 480 |
| Colonels | 1,319 |
| Total | 1,927 |

If it be estimated that for each of these officers, one surgeon and two assistant-surgeons were appointed and served in field and hospital, then the Confederate medical corps was composed of about the following:

| | |
|---|---|
| Surgeons | 1,927 |
| Assistant-Surgeons | 3,854 |
| Total | 5,781 |

This estimate places the number of surgeons and assistant surgeons at too high a figure, as may be shown by the following considerations:

*a.* Many regiments and battalions had not more than two officers.

*b.* The casualties of war were much more numerous, and promotion was much more rapid amongst the line officers than on the medical staff.

A more accurate estimate of the actual number of medical officers actively engaged in the Confederate army during the war of 1861-1865, may be based upon the number of regiments, battalions and legions of infantry, cavalry and artillery furnished by the individual States during the civil war.

| | |
|---|---|
| Total number of Regiments— | |
| Infantry | 519 |
| Cavalry | 125 |
| Artillery | 13 |
| Total | 657 |

These regiments were furnished by the individual States as follows:

| | Regiments of— Infantry. | Cavalry. | Artillery |
|---|---|---|---|
| Alabama | 57 | 3 | |
| Arkansas | 34 | 6 | |
| Florida | 9 | 3 | |
| Georgia | 65 | 11 | |
| Kentucky | 11 | 9 | |
| Louisiana | 34 | 1 | 1 |
| Maryland | 1 | | |
| Mississippi | 51 | 4 | 1 |
| Missouri | 15 | 6 | |
| North Carolina | 58 | 6 | 4 |
| South Carolina | 33 | 7 | 3 |
| Tennessee | 67 | 12 | |
| Texas | 22 | 32 | |
| Virginia | 64 | 19 | 4 |
| Confederate | 8 | 6 | |
| Total | 519 | 125 | 13 |

| | |
|---|---|
| Grand total of regiments | 657 |
| Total number of battalions— | |
| Infantry | 67 |
| Cavalry | 28 |
| Artillery | 50 |
| Total number of battalions | 145 |
| Total legions— | |
| Infantry | 13 |
| Cavalry | 3 |
| Artillery | ... |
| Total | 16 |
| Total number of battalions | 161 |
| Total regiments | 657 |
| Total regiments, battalions and legions comprising the Confederate army during the war of 1861-1865 | 818 |

If one surgeon and two assistant-surgeons be allowed to each command actively engaged in the field during the civil war of 1861-1865, the numbers would be as follows:

| | |
|---|---|
| Surgeons | 818 |
| Assistant-surgeons | 1,636 |
| | 2,454 |

The medical officers of the Confederate navy numbered:

| | |
|---|---|
| Surgeons | 22 |
| Assistant-surgeons | 10 |
| Passed assistant-surgeons | 41 |
| Total medical officers C. S. N | 73 |

If to the above be added the surgeons of the general hospitals, recruiting and conscript camps, the entire number of medical officers in the Confederate army during the war of 1861-1865 did not amount to 3,000.

The Surgeon-General of the United Confederate Veterans has endeavored to construct an accurate roster from his labors in the field and hospital during the war, and the official roll of the Confederate armies in the field, and thus far he has been able to record the names and ranks of over one thousand Confederate surgeons and assistant-surgeons.

The official list of the patrolled officers and men of the Army of Northern Virginia, surrendered by General Robert E. Lee, April 9th, 1865, furnished 310 surgeons and assistant-surgeons.

The co-operation in this most important work is solicited from every surviving members of the medical corps of the Southern Confederacy. When perfected, this roster will be published as a roll of honor and deposited in the archives of the United Confederate Veterans.

II. *The determination of the number and condition of the surviving Confederate soldiers, who were disabled by wounds and diseases received in the defense of the rights and liberties of the Southern States.*

To accomplish this important work the following inquiries have been addressed to the Governors of the Southern States, namely, Alabama, Arkansas, Florida, Georgia, Kentucky, Louisiana Mississippi, South Carolina, North Carolina, Tennessee, Texas Virginia:

(CIRCULAR NO. 2.)

OFFICE SURGEON-GENERAL UNITED CONFEDERATE VETERANS,
156 WASHINGTON AVENUE, 4TH DISTRICT,
NEW ORLEANS, LA., February 9, 1890.

*To His Excellency——— Governor——— State of———*

The attention of your Excellency is respectfully directed to the fact that in the year 1889, the Association of United Confederate Veterans was formed in New Orleans for historical, social and benevolent purposes.

Our illustrious commanding general, His Excellency, Gen. John B. Gordon, has ordered the assembling of the Confederate veterans in Chattanooga, Tenn., the 3d of July, 1890.

The welfare of the United Confederate Veterans will be materially promoted if your Excellency will furnish the Surgeon-General with the following data.

I. Number of troops furnished to the Confederate States by the State of——

II. Number of killed during the civil war of 1861–65.

III. Number of wounded during the civil war of 1861–65.

IV. Number of deaths by wounds and disease.

V. Number of Confederate survivors now living in the State of——

VI. The amount of money supplied by the State of——— for the relief and support of the survivors of the Confederate army from the close of the civil war in 1865 to the present date of 1890.

VII. Name, location and capacity of all establishments, hospitals or homes devoted to the care of the maimed, sick and indigent survivors of the Confederate States army.

VIII. A detailed statement of the money expended by the State of——— for the support of the maimed, disabled and indigent survivors of the Confederate army.

Respectfully, your obedient servant,

JOSEPH JONES,
*Surgeon-General United Confederate Veterans.*

It was earnestly desired that prompt and full reports on the part of the chief executives of the Southern States would have enabled the Surgeon-General to place in the hands of the Commanding-General of the United Confederate Veterans at the first reunion on the 4th of July, 1890, full statistics of the number of disabled Confederate veterans cared for by the individual States.

Replies have been received from only six of the thirteen States of the late Confederacy, and in three of these States it appears that no official assistance has been rendered by the State authorities to the Confederate veterans of 1861–65. The Southern States are morally bound to succor and support the men who were disabled by wounds and disease in their service, and the widows and orphans of those who fell in battle.

The Confederate soldiers who engaged in the struggle for constitutional liberty and the right of self-government were neither rebels nor traitors; they were true and brave men, who devoted their fortunes and their lives to the mothers who bore them, and their precious blood watered the hills, valleys and plains of their native States, and their bodies sleep in unknown graves, where they shall rest until the last great trump shall summon all alike, the conqueror and the conquered. The survivors have no government with its hundreds of millions for pensions. In the loneliness and suffering of advancing years and increasing infirmities, they can look alone for help to the States which they served so faithfully in battle, in victory and in defeat. The noble soldiers, who composed the illustrious armies of Northern Virginia and Tennessee, made a gallant fight against overwhelming odds for what they believed to be sacred rights and constitutional liberty. The contest was decided by the sword against them. These *matchless soldiers* accepted the issue in good faith, they returned to their homes, they resumed the avocations of peace, and engaged in building up the broken fortunes of family and country.

These brave soldiers have discharged the *obligations of good and peaceful citizens as well as they have performed the duties of thorough soldiers on the battle-field.*

It has been well said that no country ever produced braver or more intelligent and chivalric soldiers, or more industrious, law-abiding and honorable citizens than were the soldiers who surrendered with the Confederate flag. The earth has never been watered with richer blood than that shed by the noble men who fell beneath its folds. I have the honor, general, to remain,

Your obedient servant,

JOSEPH JONES, M. D.,

*Surgeon-General United Confederate Veterans.*

